# Safety and feasibility of retrograde INOUE-BALLOON for balloon aortic valvuloplasty without rapid ventricular pacing during transcatheter aortic valve replacement

**DOI:** 10.1007/s12928-021-00789-0

**Published:** 2021-06-10

**Authors:** Ryo Ninomiya, Michiko Yoshizawa, Yorihiko Koeda, Yu Ishikawa, Akiko Kumagai, Masaru Ishida, Fumiaki Takahashi, Tetsuya Fusazaki, Atsushi Tashiro, Hajime Kin, Yoshihiro Morino

**Affiliations:** 1 Division of Cardiology, Department of Internal Medicine, Iwate Medical University, 2-1-1, Idaidori, Yahaba, Iwate, 028-3694 Japan; 2 Division of Medical Engineering, Department of Information Science, Center for Liberal Arts and Sciences, Iwate Medical University, Iwate, Japan; 3 Department of Laboratory of Medicine, Iwate Medical University, Iwate, Japan; 4 Department of Cardiovascular Surgery, Iwate Medical University, Iwate, Japan

**Keywords:** Aortic stenosis, Hemodynamics, Prolonged hypotension

## Abstract

Rapid ventricular pacing (RVP) is commonly employed during transcatheter aortic valve replacement (TAVR); however, frequent TAVR is associated with worse prognoses. The retrograde INOUE-BALLOON^®^ (IB) allows balloon aortic valvuloplasty (BAV) without RVP. The aim of this study was to evaluate the feasibility of retrograde IB for TAVR preparation. The study population included 178 consecutive patients (mean age, 84 ± 5 years; male, 47%) who underwent retrograde BAV before prosthetic valve replacement via the transfemoral approach. Patients were divided into a retrograde IB group without RVP (*n* = 74) and a conventional balloon (CB) group with RVP (*n* = 104). The primary endpoint was prolonged hypotension after BAV (reduced systolic pressure < 80 mmHg for over 1 min or vasopressor drug requirement). The incidence of prolonged hypotension after BAV was significantly lower in the IB group compared with the CB group (4% vs. 16%, *p* = 0.011). Balloons were able to penetrate and expand the aortic valve in both groups. RVP was used less for total TAVR in the IB group compared with the CB group. The aortic valve area-index after BAV was not significantly different between the two groups (0.72 ± 0.14 cm^2^/m^2^ vs. 0.71 ± 0.12 cm^2^/m^2^; *p* = 0.856). Multivariate analysis demonstrated that IB use was associated with avoidance of prolonged hypotension (OR, 0.27 [0.059–0.952]; *p* = 0.041). In conclusion, BAV using retrograde IB without RVP is both safe and feasible. More stable hemodynamics were achieved using retrograde IB by avoiding RVP during TAVR.

**Supplementary Information:**

The online version contains supplementary material available at 10.1007/s12928-021-00789-0.

## Introduction

Methods for transcatheter aortic valve replacement (TAVR) are rapidly evolving with major refinements in technology, procedural techniques, patient selection, and biomedical engineering. In recent years, TAVR has become much simpler and safer, and, with expanding indications, an increasing number of younger patients are being referred for TAVR. Although TAVR has been used extensively, the development of safer and more precise procedures is essential. Rapid ventricular pacing (RVP), a common step during TAVR, is associated with prolonged hypotension and unstable hemodynamics. Frequent RVP is associated with worse prognoses [1–3]. In addition, balloon aortic valvuloplasty (BAV) before TAVR is not necessary for all patients but some patients require balloon dilation before prosthetic valve replacement.

The INOUE-BALLOON^®^ (IB) was first reported in 1984 as a device for percutaneous transvenous mitral commissurotomy (PTMC) [4]. The volume-controlled hourglass shape of the IB ensures proper positioning at the stenosis site, prevents migration of the catheter, and provides optimal dilation. Subsequently, the IB designed for PTMC was also applied for antegrade BAV and demonstrated favorable clinical outcomes [5] despite technically complex strategies. According to these clinical experiences, a modified IB (Toray Industries Inc., Tokyo, Japan) for retrograde BAV was developed and launched in Japan in 2018. The retrograde IB enables BAV without RVP during TAVR; RVP is required when using a conventional balloon [6]. Reduced intraoperative RVP using the retrograde IB was expected to further stabilize hemodynamics during TAVR. However, few reports focus on the use of the retrograde IB for TAVR [6]. Accordingly, this study was designed to verify the safety and feasibility of this new technology during the TAVR procedure.

## Methods

### Study population and design

**Fig. 1:**
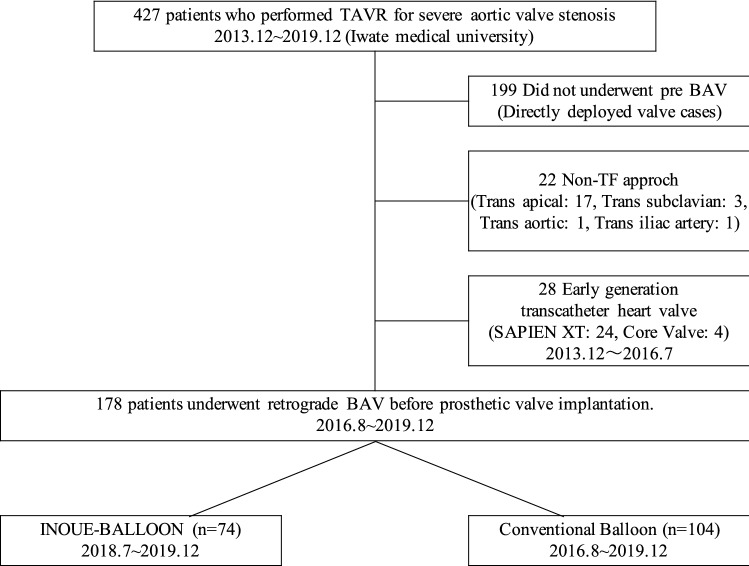
Patient recruitment flowchart

This is a single-center, retrospective, and observational study. The manufacturer of the retrograde IB, Toray, had no role in data collection, analysis, or manuscript drafting, and did not provide any financial support for the study. Consecutive patients with symptomatic severe aortic stenosis (AS) who underwent transfemoral TAVR from 2013 to 2019 at Iwate Medical University were included in the analysis (*n* = 427), retrospectively. After excluding 199 patients who did not undergo BAV, 22 patients who underwent TAVR with a non-TF approach and 28 patients who implanted early transcatheter heart valves (SAPIEN XT: 24, Core Valve: 4), 178 patients were enrolled in the study (Fig. 1). No patients underwent emergent or urgent TAVR during the study period. Eligibility for TAVR was established based on the consensus of a multidisciplinary heart team. Coronary angiography was exclusively performed before TAVR and percutaneous coronary intervention (PCI) was performed in patients who required coronary revascularization before TAVR. Regarding BAV, either the retrograde IB or the conventional balloon (CB) was used for the aortic valve; CBs included the Edwards Transfemoral Balloon Catheter (Edwards Lifesciences; Irvine), OSPYKA VACS^®^ II (Osypka AG; Germany), and Z-MED II™ (NuMED Inc.; Canada). The study protocol conforms to the ethical guidelines of the 1975 Declaration of Helsinki and prior approval by the Human Research Committee of our institution was obtained. Written informed consent for data collection was obtained from each patient before TAVR (MH2018-503).

### Retrograde INOUE-BALLOON device

**Fig. 2:**
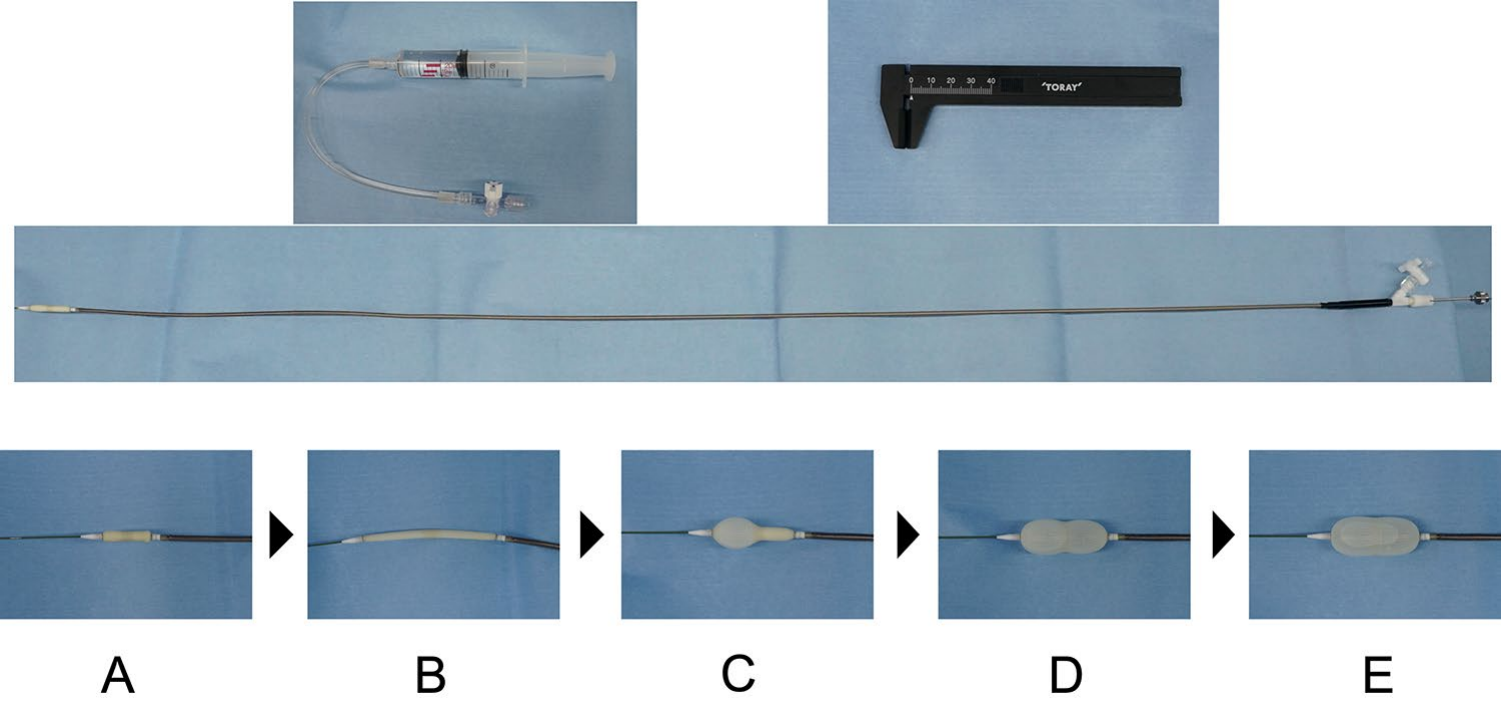
Balloon structure and expansion process. The IB catheter is an over-the-wire system with a lateral lumen for balloon dilation. Diluted contrast medium is injected with an accessory syringe to inflate the balloon. The balloon diameter is measured with the accessory caliper. While the catheter is stabilized to the aortic valve, the balloon is inflated with a prescribed amount of diluted contrast medium to dilate the aortic valve from a dumbbell to cylinder type. **A** Balloons in normal condition. **B** Balloon-extension state: Catheter insertion into the sheath. **C** Only one side of the balloon is inflated when the balloon is positioned at the aortic valve. **D** Dumbbell-shaped balloon, the central taper of which fits into the aortic valve. **E** The balloon is fully inflated during aortic valve dilation

The retrograde IB for BAV is designed to prevent the balloon from slipping through the central waist during biphasic inflation. The retrograde IB allows for balloon expansion without RVP. The IB has a long, thin shaft compatible with 0.035ʺ, and the tip of the balloon has an elliptical shape to match the rigid guidewire and calcified valve. The catheter is manufactured of polyvinyl chloride, with a balloon attached to the distal end. The balloon is composed of two latex layers, with polyester micromesh between the two layers. The catheter is 9F in diameter, and the inflated balloon length is 35 mm, which is the ideal length to prevent migration. The special inflated shape of the balloon enables the IB to minimize slipping at the aortic valve. As a result, significant dives into the left ventricle are rare, and the IB reduces the risk of balloon injury to the left ventricular muscle. Five types of balloons, with maximum expansion diameters of 20 mm, 22 mm, 24 mm, 26 mm, and 28 mm, are available. A syringe is used to manually inflate the balloon, and the balloon diameter is measured with a ruler, very similar to the traditional IB for mitral valve commissurotomy (Fig. 2). The retrograde IB has the advantages of stable fixation and multistage expansion without RVP. The balloon is characterized by reliable valve expansion as a result of its special expansion shape, in addition to speedy expansion and contraction, and gradual valve expansion.

### Definitions and outcomes

Coronary revascularization was defined as patients who had previously undergone PCI or coronary artery bypass graft (CABG). Acute kidney injury was classified according to the AKIN criteria [7]. Vascular complications, bleeding, and stroke were defined and classified by the Valve Academic Research Consortium-2 definition [8]. Balloon slippage was defined as the complete extrusion of the balloon from the aortic valve during inflation. The full disclosure of invasive blood pressure recordings during the entire TAVR procedure was reviewed retrospectively. The number of RVP, prolonged hypotension, and duration of the procedure were determined by referring to the surgical records of the anesthesiologists. RVP in this study included pacing for pre-dilatation, post-dilatation, and valve deployment. RVP for the threshold and output testing of the pacemaker were excluded.

The primary endpoint of this study was prolonged hypotension immediately after BAV. Prolonged hypotension was defined as reduced systolic pressure to < 80 mmHg for over 1 min or requirement for the administration of vasopressor drugs. All suspected events were adjudicated by a blinded interventional cardiologist. The secondary endpoints were the successful passage and dilation of the retrograde IB, the number of RVP during the procedure, and the index of aortic valve area (AVA-I) after BAV by echocardiography. Two independent and blinded observers (a cardiac ultrasound specialist and a TAVR specialist) measured the AVA-I by planimetry in the transesophageal echocardiography.

### Statistical analysis

All statistical analyses were performed using JMP^®^ 13 (SAS Institute Inc., Cary, NC, USA). Continuous variables are expressed as mean ± SD or median and interquartile range as appropriate. Qualitative variables are expressed as numbers and percentages. Normality was checked using the Shapiro–Wilk test. Differences between means were evaluated using paired and unpaired (for independent group comparisons) Student t-tests for normally distributed data. The Mann–Whitney or Wilcoxon signed-rank tests were used to evaluate non-parametric data. The chi-square test was used for categorical variables and the Fisher’s exact test was used for categorical variables with low frequencies (expected cell count < 5). Pearson correlation coefficients were used to investigate the relationship between cardiac reverse remodeling parameters and baseline parameters. A two-tailed *p* value < 0.05 was considered statistically significant. Univariate logistic regression was used to evaluate the association between high-risk categories and prolonged hypotension post-BAV. Multivariate logistic regression analysis was performed, accounting for significant independent predictors of prolonged hypotension post-BAV. The factors indicated as significant in the univariate analysis were determined as parameters for the final model in the multivariate analysis.

## Results

### Baseline characteristics

Patients were divided into two groups according to pre-BAV type: Retrograde IB (*n* = 74, 42%) or CB (*n* = 104, 58%). No significant differences were observed between the groups in terms of baseline characteristics, including age, sex, serum, history of hypertension, history of diabetes, Society of Thoracic Surgery score, New York Heart Association class 3–4, permanent pacemaker, cerebrovascular disease, and cardiac artery disease (Table 1). The numbers of patients who underwent coronary revascularization were not significantly different between the two groups. Three patients underwent CABG. Baseline echocardiography showed no significant differences in peak aortic velocity, mean pressure gradient, ejection fraction, severe aortic regurgitation (AR), and severe mitral regurgitation between the two groups, while the AVA-I was significantly smaller in the IB group.

**Table 1:** Baseline patient and procedural characteristics

	INOUE (*n* = 74)	Conventional (*n* = 104)	*p* value
Clinical characteristics			
Age, years	83 ± 5	84 ± 5	0.109
Male, *n* (%)	35 (47)	39 (38)	0.191
NYHA > III, *n* (%)	13 (18)	15 (14)	0.491
Dyslipidemia, *n* (%)	29 (39)	49 (47)	0.294
Diabetes, *n* (%)	19 (25)	25 (24)	0.803
Hypertension, *n* (%)	59 (80)	87 (84)	0.502
Chronic renal failure, *n* (%)	40 (54)	56 (54)	0.978
Atrial fibrillation, *n* (%)	12 (16)	25 (24)	0.205
Previous MI, *n* (%)	2 (3)	3 (3)	0.942
Coronary revascularization, *n* (%)	23 (31)	21 (20)	0.582
Permanent pacemaker, *n* (%)	4 (5)	6 (6)	1.000
Cerebrovascular disease, *n* (%)	12 (16)	30 (29)	0.051
COPD, *n* (%)	1 (1)	7 (7)	0.088
STS score, %	6.5 ± 3.7	5.6 ± 2.8	0.075
Clinical frailty scale	3.7 ± 0.8	3.3 ± 0.8	0.003
Laboratory data			
Hemoglobin, mg/dL	11.5 ± 1.4	11.5 ± 1.6	0.740
eGFR, mL/min/1.73m^2^	50 ± 20	54 ± 15	0.185
BNP, pg/dL	360 [18–2630]	356 [17–2834]	0.922
Echocardiographic data			
AVA-I, cm^2^/m^2^	0.43 ± 0.10	0.45 ± 0.11	0.107
Peak aortic velocity, m/s	5.1 ± 0.6	5.1 ± 0.8	0.652
Mean pressure gradient, mmHg	63 ± 17	62 ± 22	0.960
LVEF, %	62 ± 11	65 ± 8	0.021
Severe AR, *n* (%)	1 (1.4)	0 (0)	0.416
Severe MR, *n* (%)	2 (2.7)	0 (0)	0.172
Procedural data			
Balloon size, mm	19.8 ± 1.0	18.7 ± 1.8	< 0.001
RVP during BAV, *n* (%)	0 (0)	104(100)	N/A
Balloon-expandable valve, *n* (%)	46 (49)	71 (61)	0.398
Valve type			
SAPIEN 3, *n* (%)	46 (49)	71 (61)	
EvolutR/Pro, *n* (%)	28 (46)	33 (54)	

*AR* aortic regurgitation, *AVA-I* AVA indexed, *BNP* brain natriuretic peptide, *COPD* chronic obstructive pulmonary disease, *eGFR* estimated glomerular filtration rate, *LVEF* left ventricular ejection fraction, *MI* myocardial infarction, *MR* mitral regurgitation, *NYHA* New York Heart Association, *STS* Society of Thoracic Surgery

### Procedural outcomes and postoperative clinical outcomes within 30 days

The procedural outcomes immediately after BAV and TAVR are shown in Table 2. The balloons exclusively penetrated and expanded the aortic valves in both groups. None of the patients in the IB required RVP during BAV, whereas all patients in the CB group required RVP during BAV. Compared to the CB group, the IB group used fewer RVP during the TAVR procedures. After BAV, the AVA-I on the transesophageal echocardiogram was significantly enlarged in both groups (IB: 0.43 ± 0.10 cm^2^/m^2^ vs. 0.72 ± 0.14 cm^2^/m^2^, *p* < 0.001; CB: 0.45 ± 0.11 cm^2^/m^2^ vs. 0.71 ± 0.12 cm^2^/m^2^, *p* < 0.001) and the AVA-I after BAV were similar. The frequency of progression to acute severe AR after BAV was similar between the two groups. The TAVR procedure time was shorter in the IB group (62 min vs. 78 min, *p* < 0.001). Prolonged hypotension after BAV was less frequent in the IB group compared with the CB group (4% vs. 16%, *p* = 0.011). In the IB group, no cases of cardiopulmonary arrest (CPA) occurred immediately after BAV, whereas three cases of CPA occurred in the CB group. However, compared to the CB group, the IB group had a relatively higher incidence of balloon slippage due to the omission of RVP. No complications due to balloon slips were observed. Furthermore, the incidence of balloon slips did not impact clinical outcomes, with no aortic dissection in the slip group (0% vs. 0.8%, *p* = 1.000) and a stroke rate of 1.9% (1.9% vs. 2.4%, *p* = 1.000). No significant differences in maximum creatine kinase levels within 24 h postoperatively between the two groups were observed (IB vs. CB: 107 [43–482] vs. 104 [42–331], *p* = 0.953). Creatine kinase-MB tended to increase in the CB group (IB vs. CB: 14 [3–70] vs. 16 [4–69], *p* = 0.064).

**Table 2:** Procedural outcome**s**

	INOUE (*n* = 74)	Conventional (*n* = 104)	*p* value
Post-BAV			
Balloon slip, *n* (%)	36 (49)	18 (17)	< 0.001
Prolonged hypotension, n (%)	3 (4)	17 (16)	0.011
AVA-I, cm^2^/m^2^	0.72 ± 0.14	0.71 ± 0.12	0.856
Progression to acute severe AR, *n* (%)	2 (3)	2 (2)	0.731
Post-TAVR			
Procedure time, min	62 [43–485]	78 [45–355]	< 0.001
Frequency of RVP in TAVR, *n*	0.8 ± 0.7	2.0 ± 0.8	< 0.001
Total RVP time, min	15.6 ± 12.9	28.7 ± 13.5	< 0.001
Post-BAV, *n* (%)	11 (11)	13 (18)	0.178

*AR* aortic regurgitation, *AVA-I* AVA indexed, *BAV* balloon aortic valvuloplasty, *TAVR* transcatheter aortic valve replacement

Patients in the IB group showed no cases of symptomatic stroke after TAVR. Three patients died within 30 days in the CB group, but none in the IB group. The clinical outcomes after TAVR were not significantly different between the two groups (Table 3).

**Table 3:** Postoperative clinical outcome within 30 days

	INOUE (*n* = 74)	Conventional (*n* = 104)	*p* value
All-cause death, *n* (%)	0 (0)	3 (3)	0.267
Adverse events			
Minor vascular complications, *n* (%)	0 (0)	3 (3)	0.267
Major vascular complications, *n* (%)	2 (3)	0 (0)	0.172
Minor bleeding, *n* (%)	4 (5)	13 (13)	0.129
Major bleeding, *n* (%)	1 (1)	3 (3)	0.642
Life-threatening bleeding, *n* (%)	1 (1)	0 (0)	0.416
Permanent pacemaker, *n* (%)	2 (3)	6 (6)	0.472
Stroke, *n* (%)	0 (0)	2 (2)	0.512
Acute kidney injury, *n* (%)			1.000
Stage 1	13 (10)	5 (5)	
Stage 2	0 (0)	0 (0)	
Stage 3	2 (3)	1 (1)	
New atrial fibrillation, *n* (%)	3 (4)	5 (4)	1.000

### Logistic regression analysis of predictors for prolonged hypotension post-BAV

In the univariate analysis with logistic regression models, BAV with the IB, RVP > 35 ms, and history of atrial fibrillation (AF) were independent predictors of prolonged hypotension after BAV (IB: OR, 0.22 [0.061–0.768]; *p* = 0.018; RVP > 35 ms: OR, 2.84 [1.034–7.510]; *p* = 0.043; AF: OR, 2.97 [1.111–7.920]; *p* = 0.023). The multivariate analysis with logistic regression models indicated that a BAV with the IB was an independent predictor of prolonged hypotension after BAV (OR, 0.27 [0.059–0.952]; *p* = 0.041) (Table 4).

**Table 4:** Univariate and multivariate logistic regression analyses of predictors for prolonged hypotension post-BAV

	Univariate analysis			Multivariate analysis		
	OR	95% CI	*p* value	OR	95% CI	*p* value
Male	1.40	0.578–3.731	0.419			
Age	1.02	0.930–1.123	0.645			
NYHA > 3	0.91	0.250–3.367	0.896			
Hypertension	0.46	0.162–1.306	0.145			
Diabetes	2.26	0.858–5.952	0.099			
Atrial fibrillation	2.97	1.111–7.920	0.023	2.53	0.886–6.929	0.081
Coronary revascularization	1.96	0.727–5.307	0.183			
eGFR	0.99	0.964–1.019	0.517			
BNP	1.00	0.999–1.000	0.969			
LVEF (Simpson)	0.98	0.941–1.029	0.488			
AVA-I	0.77	0.032–18.946	0.137			
LVEDV	0.99	0.970–1.012	0.356			
LVESV	1.01	0.978–1.035	0.671			
Balloon size	0.98	0.941–1.029	0.488			
RVP time	1.03	0.992–1.060	0.140			
Long RVP (> 35 s)	2.84	1.034–7.510	0.043	1.59	0.534–4.594	0.396
INOUE-BALLOON without RVP	0.22	0.061–0.768	0.018	0.27	0.059–0.952	0.041

*AVA-I* AVA indexed, *eGFR* estimated glomerular filtration rate, *LVEDV* left ventricular end-diastolic volume, *LVEF* left ventricular ejection fraction, *LVESV* left ventricular end-systolic volume, *MI* myocardial infarction, *NYHA* New York Heart Association, *RVP* rapid ventricular pacing

## Discussion

The present cohort study is the first to demonstrate the safety and feasibility of the retrograde IB for pre-dilatation of TAVR. The major findings are as follows: (1) retrograde IB was successfully performed without RVP for all patients, (2) retrograde IB provided a similar capability for aortic valve expansion to CB, and (3) retrograde IB was less likely to cause systemic hypotension immediately after balloon dilation.

BAV is often required before valve replacement to facilitate passage of the valve with high calcification or a very narrow valve area. Retrograde IB was developed for BAV without RVP during TAVR. This study showed that penetration of the balloon through the stenotic aortic valve was similarly accomplished with either the IB or CB. In addition, despite the smaller baseline AVA-I with the retrograde IB, the AVA-I after pre-dilatation was almost identical.

**Fig. 3:**
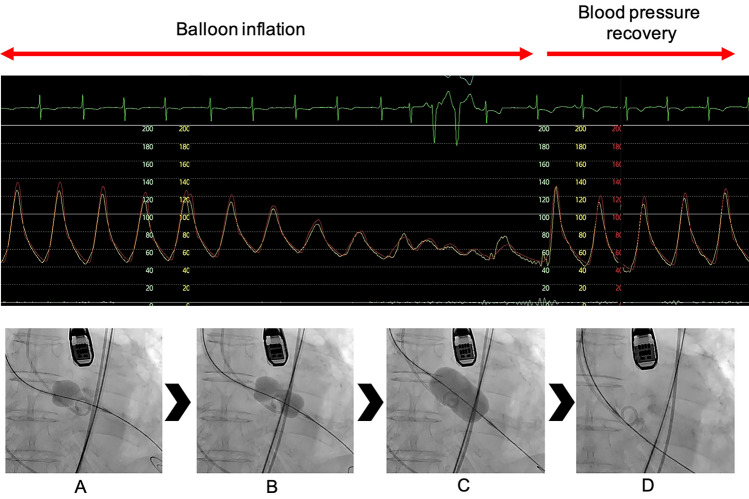
Aortic pressure during INOUE-BALLOON inflation. The balloon is inflated in three steps. Although blood pressure falls during full inflation, it recovers immediately after deflation. **A** First step: half balloon dilation; there is no decrease in blood pressure. **B** Second step: dumbbell-shaped balloon expansion; the aortic valve is anchored, and the blood pressure gradually decreases. **C** Third step: full balloon dilation and change to transient low pulse pressure. **D** Balloon deflation: the blood pressure immediately recovers to the baseline level after inflation

RVP is necessary for BAV with a CB [9, 10]. However, it is well known that RVP decreases microvascular tissue perfusion [11] and leads to right ventricular dysfunction [12]. Furthermore, RVP is associated with myocardial damage [3, 13, 14], prolonged hypotension, and unfavorable prognoses [1, 15, 16]. In contrast, stable balloon fixation and multistage expansion with an hourglass shape, even without RVP, ensures that the calcified aortic valve is opened and prevents the collapse of hemodynamics (Fig. 3). In the present study, the low frequency of prolonged hypotension after expansion with retrograde IB may be strongly associated with the absence of RVP. The retrograde IB group had significantly lower total RVP during TAVR, which may have increased intraoperative hemodynamic stability. The clinical implication of the use of IB is that it reduces intraoperative anxiety factors by avoiding prolonged hypotension, leading to a safer TAVR procedure. Moreover, stroke events and 30-day deaths are more commonly associated with the valve replacement procedure than balloon pre-dilatation. The decrease in the total number of RVP and the hemodynamic stability after pre-dilatation may potentially improve clinical outcomes in the IB group. Moreover, in this study, prolonged hypotension after BAV was observed in many patients with low-flow, low-gradient aortic stenosis (LFLG AS), suggesting that LFLG AS is prone to hemodynamic instability after BAV. In patients with LFLG AS, IB may be used to safely prepare for valve implantation. However, further investigation in more patients is needed.

The higher frequencies of balloon slippage during expansion in the IB group, primarily caused by the omission of RVP, did not appear to be problematic, and no impact on hemodynamic stability or left ventricular injury was observed in this study. However, myocardial damage due to balloon dives into the left ventricle and vascular damage, including aortic dissection due to slippage into the aortic side, should be monitored. As a result of our experiences with the retrograde IB, we developed specific tips to prevent the IB from slipping, including anchoring the initially inflated portion of the balloon to the native aortic balloon (push or pull the balloon slightly to fix). Furthermore, both the proximal and distal portions can be inflated earlier, which might help to determine the initial longitudinal balloon position relative to the native aortic valve.

In addition to the selection of the BAV balloon, this study indicated that history of atrial fibrillation (AF) is independently associated with prolonged depression of blood pressure after BAV. In terms of the pre-existing risk of AF, the mechanisms used to prevent prolonged hypotension immediately after BAV are unclear. Atrial kick is defined as the force produced by the atria contracting before ventricular systole or at the end of ventricular diastole. In patients with AF, cardiac output is reduced by the loss of atrial kick [17]. The contribution of the atrial kick depends on the heart rate and heart structure. During tachycardia, the ventricular diastole period is reduced leading to increased dependence of the ventricular filling on the atrial kick. Patients with increased ventricular stiffness and decreased relaxation function (ventricular diastolic dysfunction) have decreased rapid filling and early diastole and become more dependent on atrial kick [18]. Most patients with AS show left ventricular hypertrophy, impaired relaxation, and reduced left ventricular capacity. Therefore, patients with AS might be more likely to have prolonged hypotension immediately after RVP. Furthermore, recent studies suggest that AF is associated with impaired baroreflex and that restoration of sinus rhythm improves baroreflex gain [19]. In addition, a long RVP time may also prolong the time of myocardial blood flow reduction and delay myocardial recovery after RVP. RVP may have a temporary adverse effect on the myocardium [12, 20]. Furthermore, prolonged RVP has been reported to worsen prognosis after TAVR [1]. RVP is an inseparable tool for TAVR, but frequent and prolonged RVP should be avoided. The current experience with IB remains limited and further research is required to establish its advantages, as well as to share tips and tricks for the optimal use of this technology.

Our study has several limitations. First, this is a relatively small, single-center, retrospective study of patients in a relatively high-volume center. Future studies with larger sample sizes are needed and should ideally be conducted in a randomized fashion. Second, the enrollment periods of the two groups were different; the retrograde IB was predominantly used in the second half of the study because it has been used since 2018. Furthermore, following the accumulation of experience, TAVR devices are likely to improve, which might impact the clinical outcomes at each time point. The statistically significant differences in procedural times between the two groups may be due to differences in hemodynamic conditions during pre-dilatation; however, these results may also be affected by potential differences in technical skills and experience of the heart team between the enrollment periods. Finally, there is no established rule to determine the balloon size; however, all balloon sizes were determined following an extensive discussion between the members of the experienced heart team, considering aortic valve measurements and the amount of calcification detected on cardiac computed tomography scan and echocardiography. The balloon size required for the preparation of the prosthetic valve implantation was usually selected.

In conclusion, BAV using retrograde IB without RVP is both safe and feasible. Furthermore, the use of retrograde IB has led to more stable hemodynamics by omitting the need for RVP during TAVR.

## Supplementary Information

Below is the link to the electronic supplementary material.

Supplementary file1 (DOCX 18 KB)

## Abbreviations

AF: Atrial fibrillation
AR: Aortic regurgitation
AS: Aortic stenosis
AVA-I: Aortic valve area-index
BAV: Balloon aortic valvuloplasty
CPA: Cardiopulmonary arrest
CABG: Coronary artery bypass graft
CB: Conventional balloon
IB: INOUE-BALLOON
PTMC: Percutaneous transvenous mitral commissurotomy
RVP: Rapid ventricular pacing
TAVR: Transcatheter aortic valve replacement
