# Correction to: Safety and feasibility of retrograde INOUE‑BALLOON for balloon aortic valvuloplasty without rapid ventricular pacing during transcatheter aortic valve replacement

**DOI:** 10.1007/s12928-021-00816-0

**Published:** 2021-11-12

**Authors:** Ryo Ninomiya, Michiko Yoshizawa, Yorihiko Koeda, Yu Ishikawa, Akiko Kumagai, Masaru Ishida, Fumiaki Takahashi, Tetsuya Fusazaki, Atsushi Tashiro, Hajime Kin, Yoshihiro Morino

**Affiliations:** 1 Division of Cardiology, Department of Internal Medicine, Iwate Medical University, 2-1-1, Idaidori, Yahaba, Iwate, 028-3694 Japan; 2 Division of Medical Engineering, Department of Information Science, Center for Liberal Arts and Sciences, Iwate Medical University, Iwate, Japan; 3 Department of Laboratory of Medicine, Iwate Medical University, Iwate, Japan; 4 Department of Cardiovascular Surgery, Iwate Medical University, Iwate, Japan

## Correction to: Cardiovascular Intervention and Therapeutics 10.1007/s12928-021-00789-0

In the original publication of the article, in table 1, there was an error in the number of conventional balloons used. This was because we had originally enrolled 132 patients and the entry was left out. The study ended up including 104 patients who used the Conventional Balloon. The 100% rate remains the same because rapid pacing is still being done in all patients.

**Table 1:** Baseline patient and procedural characteristics

	Page	Line	Error	Correction
#1	5	33 (Table 1)	132 (100)	104 (100)
#2	7	21 (Table 4)	Long RVP (> 35 ms)	Long RVP (> 35 s)
#3	8	50	The authors are deeply grateful to the laboratory members Yumiko Okuyama (research nurse), Kayoko Fujiwara, and Kanako Omiya (secretaries)	The authors are deeply grateful to the laboratory members Yumiko Okuyama (research nurse), Kayoko Fujiwara, and Kanako Omiya (secretaries). This work was supported by JSPS KAKENHI Grant Number JP 20K17093

In Table 4, Long RVP is 35 s is the correct information.

**Table 4:** Univariate and multivariate logistic regression analysis of predictors for prolonged hypotension post BAV

	Univariate analysis			Multivariate analysis		
	OR	95% CI	*p* value	OR	95% CI	*p* value
Male	1.40	0.578–3.731	0.419			
Age	1.02	0.930–1.123	0.645			
NYHA > 3	0.91	0.250–3.367	0.896			
Hypertension	0.46	0.162–1.306	0.145			
Diabetes	2.26	0.858–5.952	0.099			
Atrial fibrillation	2.97	1.111–7.920	0.023	2.53	0.886–6.929	0.081
Coronary revascularization	1.96	0.727–5.307	0.183			
eGFR	0.99	0.964–1.019	0.517			
BNP	1.00	0.999–1.000	0.969			
LVEF (Simpson)	0.98	0.941–1.029	0.488			
AVA-I	0.77	0.032–18.946	0.137			
LVEDV	0.99	0.970–1.012	0.356			
LVESV	1.01	0.978–1.035	0.671			
Balloon size	0.98	0.941–1.029	0.488			
RVP time	1.03	0.992–1.060	0.140			
Long RVP (> 35 s)	2.84	1.034–7.510	0.043	1.59	0.534–4.594	0.396
INOUE-BALLOON without RVP	0.22	0.061–0.768	0.018	0.27	0.059–0.952	0.041

*AVA-I* AVA indexed, *eGFR* estimated glomerular filtration rate, *LVEDV* left ventricular end-diastolic volume, *LVEF* left ventricular ejection fraction, *LVESV* left ventricular end-systolic volume, *MI* myocardial infarction, *NYHA* New York Heart Association, *RVP* rapid ventricular pacing

In the original publication of the article, the acknowledgements section published incorrectly. The correct acknowledgements is given in this correction.

**Acknowledgements**

This work was supported by JSPS KAKENHI Grant Number JP 20K17093. The authors are deeply grateful to the laboratory members Yumiko Okuyama (research nurse), Kayoko Fujiwara, and Kanako Omiya (secretaries).

The original article has been corrected.

