# Supplementary material for: Safety and feasibility of retrograde INOUE-BALLOON for balloon aortic valvuloplasty without rapid ventricular pacing during transcatheter aortic valve replacement
Source: Cardiovasc Interv Ther. 2021 Jun 10;37(2):372–80. doi: 10.1007/s12928-021-00789-0 (PMC8927037; doi:10.1007/s12928-021-00789-0)
Supplement: Supplementary file 1 — Supplementary file1 (DOCX 18 KB) [file 12928_2021_789_MOESM1_ESM.docx]

**TABLE.** Relationship between balloon slips and clinical outcomes (supplemental)

|  | Slip (n = 54 ) | No Slip (n = 124 ) | p value |
| --- | --- | --- | --- |
|  | 1 (1.9) | 6 (4.8) | 0.677 |
| All cause death, n (%) | 0(0) | 3 (2.4) | 0.554 |
| Progression to acute severe AR, n (%) | 1 (1.9) | 3 (2.4) | 1.000 |
| VF/PCPS, n (%) | 0 (0) | 2 (1.6) | 1.000 |
| Cerebral infarction, n (%) | 1 (1.9) | 3 (2.4) | 1.000 |
| Aortic dissection, n (%) | 0 (0) | 1 (0.8) | 1.000 |

AR, aortic regurgitation; VF, ventricular fibrillation; PCPS, percutaneous cardiopulmonary support

**TABLE.** Postoperative clinical outcome within 30 days by RVP time (supplemental)

|  | RVP time (msec) | | |  |
| --- | --- | --- | --- | --- |
|  | 0–25 (n = 88) | 25–35 (n = 52) | > 35 (n = 38) | p value |
| All cause death, n (%) | 2 (2) | 0 (0) | 1 (3) | 0.527 |
| **Adverse events** |  |  |  |  |
| Minor vascular complications, n (%) | 2 (2) | 0 (0) | 1 (3) | 0.527 |
| Major vascular complications, n (%) | 1 (1) | 2 (4) | 0 (0) | 0.320 |
| Minor bleeding, n (%) | 2 (2) | 0 (0) | 2 (5) | 0.250 |
| Major bleeding, n (%) | 6 (7) | 9 (17) | 2 (5) | 0.075 |
| Life-threatening bleeding, n (%) | 0 (0) | 1 (2) | 0 (0) | 0.296 |
| Permanent pacemaker, n (%) | 3 (3) | 2 (4) | 3 (8) | 0.518 |
| Stroke, n (%) | 2 (2) | 0 (0) | 0 (0) | 0.356 |
| Acute kidney injury, n (%) |  |  |  | 0.851 |
| Stage 1 | 7 (8) | 3 (6) | 3 (8) |  |
| Stage 2 | 0 (0) | 0 (0) | 0 (0) |  |
| Stage 3 | 2 (2) | 1 (2) | 0 (0) |  |
| New atrial fibrillation, n (%) | 6 (9) | 1 (2) | 1 (3) | 0.330 |

RVP, rapid ventricular pacing

**TABLE.** Maximum cardiac enzymes levels after BAV with INOUE-BALLOON and conventional balloon (supplemental)

|  | INOUE (n = 74 ) | Conventional (n = 104 ) | p value |
| --- | --- | --- | --- |
| CK (IU/U) | 107 [43–482] | 104 [42–331] | 0.953 |
| CK-MB (IU/U) | 14 [3–70] | 16 [4–69] | 0.064 |

CK, creatine kinase; CK-MB, creatine kinase-myocardial band
